# Supplementary material for: Knowledge and practices of modern contraceptives among religious minority (Muslim) women: A cross-sectional study from Southern Nepal
Source: PLoS One. 2022 Dec 12;17(12):e0278899. doi: 10.1371/journal.pone.0278899 (PMC9744303; doi:10.1371/journal.pone.0278899)
Supplement: Attachment — Submitted filename: PONE-D-22-08164_review.pdf [file pone.0278899.s002.pdf]

PLOS ONE

Knowledge and practices of modern contraceptive among religious minority (Muslim) women: A cross-sectional study from Southern Nepal

--Manuscript Draft--

**Manuscript Number:**	PONE-D-22-08164
**Article Type:**	Research Article
**Full Title:**	Knowledge and practices of modern contraceptive among religious minority (Muslim) women: A cross-sectional study from Southern Nepal
**Short Title:**	Knowledge and practices of modern contraceptive among religious minority (Muslim) women
**Corresponding Author:**	Dipendra S Thakuri<br>Save the Children<br>Surkhet, Karnali State NEPAL
**Keywords:**	Knowledge; Practice; Modern Contraceptive Methods; Family Planning; Muslim Women; Nepal
**Abstract:**<br><br>why do another study on practice if already know the practice is low in Nepal from low minority?	Background<br><br>Uptake of family planning (FP) services could prevent many unwanted pregnancies, unsafe abortions and avert maternal deaths. Women, especially from ethnic and religious minorities, have low practice of contraceptives in Nepal. This study examined the knowledge and practices of modern contraceptive methods among Muslim women in Nepal.<br><br>Methods<br><br>A cross-sectional study was conducted among 400 Muslim women in Khajura Rural Municipality of Banke district. Data were collected using face to face semi-structured interviews. The status of knowledge and practice of modern contraceptive methods were analysed by different socioeconomic factors.<br><br>Results<br><br>Almost two thirds (69.2%) of respondents had good knowledge of modern contraceptive methods and 47.3% practised modern contraceptive methods. Women of nuclear family (adjusted odds ratio (aOR)=0.60; 95% CI: 0.38,0.95), and who work in agricultural sector (aOR=0.38; 95% CI: 0.22, 0.64) were less likely to have good knowledge on modern contraceptives. Women with primary (aOR =2.59; 95% CI: 1.43, 4.72), secondary and above education (aOR=4.41; 95% CI:2.02,9.63), women with good knowledge of modern contraceptives (aOR=2.73; 95% CI: 1.66, 4.51), who received FP counselling at health facility (aOR=4.40; 95% CI: 2.58, 7.50) had higher odds of modern contraceptives practices.<br><br>Conclusion<br><br>Muslim women had low practices despite having satisfactory knowledge of modern contraceptive methods. There is a need for more equitable and focused high-quality FP practices. Targeted interventions are needed to increase the knowledge and practices of contraceptives in the Muslim community. Such interventions include mobilisation of health workers (HWs) from their community, awareness on contraceptive methods embedding with values and culture of the Muslim religion.
**Order of Authors:**	Dipendra S Thakuri
	KC Yamuna
	Rajendra Karkee
	Resham B Khatri
**Additional Information:**	

Powered by Editorial Manager® and ProduXion Manager® from Aries Systems Corporation

Question	Response
**Financial Disclosure**<br><br>Enter a financial disclosure statement that describes the sources of funding for the work included in this submission. Review the submission guidelines for detailed requirements. View published research articles from *PLOS ONE* for specific examples.<br><br>This statement is required for submission and **will appear in the published article** if the submission is accepted. Please make sure it is accurate.<br><br>**Unfunded studies**<br>Enter: *The author(s) received no specific funding for this work.*<br><br>**Funded studies**<br>Enter a statement with the following details:<br>• Initials of the authors who received each award<br>• Grant numbers awarded to each author<br>• The full name of each funder<br>• URL of each funder website<br>• Did the sponsors or funders play any role in the study design, data collection and analysis, decision to publish, or preparation of the manuscript?<br>• **NO** - Include this sentence at the end of your statement: *The funders had no role in study design, data collection and analysis, decision to publish, or preparation of the manuscript.*<br>• **YES** - Specify the role(s) played.<br><br>* typeset	The authors received no specific funding for this work.
**Competing Interests**<br><br>Use the instructions below to enter a competing interest statement for this submission. On behalf of all authors, disclose any competing interests that could be perceived to bias this work—acknowledging all financial support and any other relevant financial or non-financial competing interests.	The authors have declared that no completing interests exist.

This statement is required for submission and **will appear in the published article** if the submission is accepted. Please make sure it is accurate and that any funding sources listed in your Funding Information later in the submission form are also declared in your Financial Disclosure statement.<br><br>View published research articles from *PLOS ONE* for specific examples.<br><br>**NO authors have competing interests**<br><br>Enter: *The authors have declared that no competing interests exist.*<br><br>**Authors with competing interests**<br><br>Enter competing interest details beginning with this statement:<br><br>*I have read the journal's policy and the authors of this manuscript have the following competing interests: [insert competing interests here]*<br><br>* typeset	
**Ethics Statement**<br><br>Enter an ethics statement for this submission. This statement is required if the study involved:<br><br>• Human participants<br>• Human specimens or tissue<br>• Vertebrate animals or cephalopods<br>• Vertebrate embryos or tissues<br>• Field research<br><br>Write "N/A" if the submission does not require an ethics statement.<br><br>General guidance is provided below. Consult the submission guidelines for detailed instructions. **Make sure that all information entered here is included in the Methods section of the manuscript.**	Ethical approval was obtained from the ethical review board of Nepal Health Research Council and educational and administrative ethical committee, faculty of Nursing and Medical College of Xi'an Jiaotong University, China, for this study. Before the collection of data, written permission was obtained from the local administrative authority Khajura Rural Municipality of Banke district.

Powered by Editorial Manager® and ProduXion Manager® from Aries Systems Corporation

**Format for specific study types**<br><br>**Human Subject Research (involving human participants and/or tissue)**<br>• Give the name of the institutional review board or ethics committee that approved the study<br>• Include the approval number and/or a statement indicating approval of this research<br>• Indicate the form of consent obtained (written/oral) or the reason that consent was not obtained (e.g. the data were analyzed anonymously)<br><br>**Animal Research (involving vertebrate animals, embryos or tissues)**<br>• Provide the name of the Institutional Animal Care and Use Committee (IACUC) or other relevant ethics board that reviewed the study protocol, and indicate whether they approved this research or granted a formal waiver of ethical approval<br>• Include an approval number if one was obtained<br>• If the study involved *non-human primates*, add *additional details* about animal welfare and steps taken to ameliorate suffering<br>• If anesthesia, euthanasia, or any kind of animal sacrifice is part of the study, include briefly which substances and/or methods were applied<br><br>**Field Research**<br><br>Include the following details if this study involves the collection of plant, animal, or other materials from a natural setting:<br>• Field permit number<br>• Name of the institution or relevant body that granted permission	
**Data Availability**<br><br>Authors are required to make all data underlying the findings described fully available, without restriction, and from the time of publication. PLOS allows rare exceptions to address legal and ethical concerns. See the PLOS Data Policy and FAQ for detailed information.	Yes - all data are fully available without restriction

Powered by Editorial Manager® and ProduXion Manager® from Aries Systems Corporation

A Data Availability Statement describing where the data can be found is required at submission. Your answers to this question constitute the Data Availability Statement and **will be published in the article**, if accepted.<br><br>**Important:** Stating 'data available on request from the author' is not sufficient. If your data are only available upon request, select 'No' for the first question and explain your exceptional situation in the text box.<br><br>Do the authors confirm that all data underlying the findings described in their manuscript are fully available without restriction?	
**Describe where the data may be found in full sentences. If you are copying our sample text, replace any instances of XXX with the appropriate details.**<br><br>• If the data are **held or will be held in a public repository**, include URLs, accession numbers or DOIs. If this information will only be available after acceptance, indicate this by ticking the box below. For example: *All XXX files are available from the XXX database (accession number(s) XXX, XXX.).*<br>• If the data are all contained **within the manuscript and/or Supporting Information files**, enter the following: *All relevant data are within the manuscript and its Supporting Information files.*<br>• If neither of these applies but you are able to provide **details of access elsewhere**, with or without limitations, please do so. For example:<br><br>*Data cannot be shared publicly because of [XXX]. Data are available from the XXX Institutional Data Access / Ethics Committee (contact via XXX) for researchers who meet the criteria for access to confidential data.*<br><br>*The data underlying the results presented in the study are available*	The data of this study can be obtained upon request to first author.

Powered by Editorial Manager® and ProduXion Manager® from Aries Systems Corporation

from (include the name of the third party and contact information or URL).<br>• This text is appropriate if the data are owned by a third party and authors do not have permission to share the data.<br><br>* typeset	
Additional data availability information:	

Powered by Editorial Manager® and ProduXion Manager® from Aries Systems Corporation

Manuscript

Click here to access/download;Manuscript;FP Manuscript docx..docx

Knowledge and practices of modern contraceptive among religious minority (Muslim) women: A cross-sectional study from Southern Nepal

Dipendra S Thakuri*[1], Yamuna KC[2], Rajendra Karkee[3], Resham B Khatri[4,5]

[1] Save the Children, Nepal Country Office, Nepal

[2] Bheri Nursing College, Purbanchal University, Nepalgunj, Nepal

[3] School of Public Health and Community Medicine, BP Koirala Institute of Health Sciences, Dharan, Nepal

[4] School of Public Health, Faculty of Medicine, University of Queensland, Brisbane, Australia

[5] Health Social Science and Development Research Institute, Kathmandu, Nepal

*Corresponding author (E-mail): dipendrathakuri@gmail.com

Abstract

Background

Uptake of family planning (FP) services could prevent many unwanted pregnancies, unsafe abortions and avert maternal deaths. Women, especially from ethnic and religious minorities, have low practice of contraceptives in Nepal. This study examined the knowledge and practices of modern contraceptive methods among Muslim women in Nepal.

Methods

A cross-sectional study was conducted among 400 Muslim women in Khajura Rural Municipality of Banke district. Data were collected using face to face semi-structured interviews. The status of knowledge and practice of modern contraceptive methods were analysed by different socioeconomic factors.

Results

Almost two thirds (69.2%) of respondents had good knowledge of modern contraceptive methods and 47.3% practised modern contraceptive methods. Women of nuclear family (adjusted odds ratio (aOR)=0.60; 95% CI: 0.38,0.95), and who work in agricultural sector (aOR=0.38; 95% CI: 0.22, 0.64) were less likely to have good knowledge on modern contraceptives. Women with primary (aOR =2.59; 95% CI: 1.43, 4.72), secondary and above education (aOR=4.41; 95% CI:2.02,9.63), women with good knowledge of modern contraceptives (aOR=2.73; 95% CI: 1.66, 4.51), who received FP counselling at health facility (aOR=4.40; 95% CI: 2.58, 7.50) had higher odds of modern contraceptives practices.

Conclusion

Muslim women had low practices despite having satisfactory knowledge of modern contraceptive methods. There is a need for more equitable and focused high-quality FP practices. Targeted interventions are needed to increase the knowledge and practices of contraceptives in the Muslim community. Such interventions include mobilisation of health workers (HWs) from their community, awareness on contraceptive methods embedding with values and culture of the Muslim religion.

Keywords: Knowledge, Practice, Modern Contraceptive Methods, Family Planning, Muslim Women, Nepal

Introduction

Family planning (FP) is one of the high impact interventions that prevent unintended pregnancies, unsafe abortions, reduce high-risk births, avert maternal and neonatal deaths, and protect women and children's health [1–4]. Despite multiple benefits, many women in need of FP methods, cannot access the FP services. This unmet need for FP results in approximately 539,000 annual unintended pregnancies in Nepal [5,6]. These unintended pregnancies can pose serious health risks to mothers and their newborns, including deaths [7]. The risks of maternal morbidity and mortality are also high among poor, rural women who have been facing many barriers in access to FP services in Nepal [5,8,9]. One in 200 women dies from pregnancy-or delivery-related causes in their lifetime in Nepal [10].

Nepal made considerable health services access and improved maternal and child health services coverage over the last three decades [11,12]. However, the FP program has poor performance and has low and stagnant progress in contraceptive prevalence rate (CPR) [13]. The Nepal Demographic Health Survey (NDHS) 2016 [12] revealed that CPR for modern contraceptive methods in Nepal was 43%, with 24% unmet need. Women from the poorest households, living in remote areas, disadvantaged ethnicities, religious minorities, and those with no education had poor knowledge and the lowest practice of contraceptive methods [14]. Many health system factors have contributed to poor progress on practices of contraceptive methods, including poor access to contraceptive methods, lack of contraceptive methods in health facilities [15], poor uptake due to perceived side effects, lack of proper counselling services on contraceptive methods and religious and cultural beliefs and value system [16,17]. Muslim women have low CPR (25.4%), high unmet need (37%) for modern contraceptive methods, high fertility and large family size in Nepal [18,19]. In Nepal, the total fertility rate has increased from 4.6 (2006) to 4.9 (2011) in Muslim populations [18]. Muslim groups had unintended pregnancies leading to the highest maternal mortality ratio (318 per 100000 live births) in Nepal [20] which suggests the need for quality FP services delivery and utilisation among Muslims.

Past evidence showed the knowledge and attitude contributed to the utilisation of modern contraceptive methods [21]. Other socioeconomic and demographic determinants were also identified as determinants of contraceptive methods such as women’s age, education, number and sex of children, occupation, and access to a health facility [3,17]. However, limited evidence

available on the status of knowledge and practices of modern contraceptive methods and their associated determinants among Muslim women in Nepal. Therefore, we aimed to address research gaps among Muslim women of Mid-western Nepal. The findings of this study could inform policymakers and program managers to design contextual policies and programmatic strategies for universal coverage of contraceptive methods among the Muslim population.

Objectives are not clear. Justification also need detailing. Why the need to look into current practice

Policy and services delivery context of FP in Nepal

Family planning program is the oldest public health program in Nepal [19], and FP services are available at the community level through Female Community Health Volunteers. Nepal's health policy 2019 and strategies also emphasised the FP program and ensuring quality FP services. Current periodic strategies and plans, such as Nepal Health Sector Strategy (NHSS) 2015-2020 [22], and the Population Perspective Plan (2010-2031) [23], have highlighted FP as the major component of the Safe Motherhood Initiative in Nepal [24]. The FP Costed Implementation Plan 2015-2021 has also highlighted the cost and implementation strategies [25]. However, these policies and programs approaches are implemented one-size-fits-all approach [19]. There have not been focused and context-specific implementation strategies to recognise religious and cultural consideration for addressing FP needs of marginalised populations.

In Nepal, modern contraceptive services provided from different outlets ranging from community to tertiary level (Figure 1). Services outlets include community clinics, health posts, static health clinics, and mobile health camps from different public, private, and private non-profit sector health institutions. In addition, several short-term modern contraceptives are available at peripheral facilities. In contrast, long-term modern contraceptives are being provided in health posts (HP), primary health care centers (PHCC) and hospitals [26].

How are the Family planning accessibility of the minority differs from the general population?

……………Insert Figure 1 here……………………

Figure 1: Types and delivery outlets of modern contraceptives in Nepal. Figure developed by authors based on information obtained from Nepal annual health report 2019[26].

Methods

Muslims in Nepal

The Muslim community is a religious minority, socially excluded, and disadvantaged group in Nepal [19], consisting of 4.4% of the total population [27]. Most of them live in Terai districts Banke, Rautahat, Kapilvastu, Parsa, Mahottari, Bara and Sunsari. A smaller proportion resides in some hills and mountain districts Achham and Arghakhanchi of Nepal. Muslims are economically, socially, educationally, and politically backwards and deprived of various facilities, including health services [28]. As a result, they have one of the least human development index (HDI) of 0.41[29] and the highest MMR [8].

Study design and setting

How are they deprived from the other population?

A community-based cross-sectional study was carried out between June and September 2019 in Khajura Rural Municipality of Banke district. The study population was married Muslim women with reproductive age of 15 to 49 years. The Khajura Rural Municipality was selected purposively. In this municipality more than one in four (26.7%) people belong to Muslim backgrounds [27]. The total population of 50,961 of Khajura Rural Municipality lives in 10,288 households. 27,457 were females, including 19,397 of reproductive age (15 to 49 years) group [27]. Four wards (of eight wards) of the municipality were selected randomly for the household survey. There was an estimated total of 1,750 Muslim married women of reproductive age (MWRA) in those selected wards [30].

Need to clarify where is the ward? Is it hospital setting?

Sampling and participants selection

The sampling frame of this study was married Muslim women aged 15-49 years. The sampling frame of Muslim MWRA was obtained from the selected ward office. Sample size was calculated using formula N=Z2pq/d2 where [Z=1.96, p=0.44 q=0.56), d =0.05] and 44% prevalence rate [31]. We determined 379 as the minimal sample size. Considering non-response rate of 10%, a sample of 400 Muslim women were interviewed among 1,750 Muslim MWRA. We selected participant through a systematic random sampling method. The first women were selected randomly, and then every fourth (having a gap of three) women were selected for the interview. If there was more than one MWRA in the family, the youngest women were included in the study. Likewise, the adjoining households were recruited if the participants were not available in the selected households.

sample size calculation needs to be base on the two DVs ;
practice and knowledge.Author only cxalculatedbased on the practice.
By right, need to use the two sample proportion sample size calculation. The author should demonstrate the highest sample size to ensure sufficient power.

Conceptual framework of the study

Figure 2 illustrates the determinants of knowledge and practices of modern contraceptive methods. We adopted and revised the conceptual framework developed by Abebe Gizaw and colleague (2011) [32]. The framework comprises women's demographic, socioeconomic and socio-cultural characteristics and characteristics of partner and other mediating factors. The framework conceptualises the relationship between different sociodemographic, socioeconomic, and socio-cultural determinants and knowledge and practices of modern contraceptive methods.

…...……………Insert Figure 2 here ………………

Figure 2: The conceptual framework of the study adapted and revised from the study by Abebe Gizaw., et al., (2011)[32]

Study variables

Based on previous studies in Nepal and elsewhere [19,33,34], explanatory variables were basic socioeconomic and demographic variables. Demographic factors were respondent's age (≤18 years,19-29 years and ≥30 years), parity (0 to 2 and ≥3), respondent's family type (nuclear and joint family) [35]. Socioeconomic variables were respondent's education (illiterate those cannot read and write, basic education, secondary and above), respondent's occupation (agriculture, daily wage workers and housewives). Similarly, husband's occupation (agriculture, business and service, daily wages worker and foreign migrant worker) and family monthly income (≤20000 NRs and >20000 NRs (120 Nepalese Rupees=1 USD, 2022). Additionally, access to FP service variable included: health facility visits for FP counselling (yes/no). Knowledge of modern contraceptive methods was also included as the independent variable for practices of modern contraceptive methods. Based on ten knowledge-based questions, a composite measure of knowledge was created.

Outcome variables

Two outcome variables were included: knowledge on modern contraceptive methods (good and poor knowledge), and practices of modern contraceptive methods (yes or no). Knowledge on modern contraceptive methods was created using 10 sets of questions related to modern contraceptives such as ever heard about family planning (yes/no), female sterilisation is one way to avoid pregnancy (yes/no), oral contraceptive pills do not guarantee 100% protection (yes/no), women using the birth control injectable must get an injection every three months (yes/no), using both a condom and the pills is considered to be very effective (yes/no), use of contraceptive prevents unwanted pregnancies (yes/no), contraceptive methods are appropriate to space childbirths (yes/no), condom provides dual protection (prevents STI/HIV and unplanned pregnancies) (yes/no), contraceptive education is important for women who want to use contraception (yes/no), and common side effects of contraceptive pills include mood swings and weight gain (yes/no). Knowledge of modern contraceptive methods was then scored by assigning one point for each correct response. We considered a score of mean and above 'Good Knowledge' and a score of below mean 'Poor Knowledge'[7,36]. To assess the practice of modern contraceptive methods, women were asked if they used modern contraceptive methods in the last six months prior to this survey and coded their response as 'yes' or 'no'.

Data collection tools and techniques

WHo translate and how is the process?

A questionnaire on knowledge and practice of modern contraceptive methods were adopted from the previous studies [19,31,37] and survey [12]. The structured questionnaire was first developed in English, then translated into Nepali and local language (Awadhi). It was pretested among 20 women aged 15-49 years in adjoining ward to refine it. Necessary adjustments were made, including in the flow of questions patterns and language style. The local language was used in data collection. A face-to-face interview was conducted in participant's households. The interview was carried out in a separate area of participants' households to ensure confidentiality. Participation was voluntary, and none approached respondents refused to be interviewed. Data were collected by local enumerators consisting of 3 females. The enumerators were the local Muslim community. They were recruited based on their educational background, local language knowledge, and prior data collection experience. The two days training was provided to the

enumerators about the study purpose, methodologies, tools, and techniques before preceding the actual data collection. All the data collection related field activities were closely supervised and monitored by the second author (YKC).

Data analysis

Data analysis was performed using SPSS version 25.0 (SPSS Inc., Chicago, IL). The collected data were entered, coded, and cross-checked to ensure consistency. Descriptive analyses were employed and reported as frequencies and proportions. The Chi-square test was conducted to assess the association between independent and outcome variables. Binomial logistic regression was examined to identify the determinants of knowledge and practices of modern contraceptive methods. Odds ratio with 95% confidence interval (CIs) were reported. The significance level was set at $p < 0.05$ (two-tailed).

Ethical approval

Ethical approval was obtained from the ethical review board of Nepal Health Research Council and educational and administrative ethical committee, faculty of Nursing and Medical College of Xi'an Jiaotong University, China, for this study. Before the collection of data, written permission was obtained from the local administrative authority Khajura Rural Municipality of Banke district. Before the interview process, enumerators and the second author (YKC) met Muslim religious leaders, shared the objective of the study, and obtained their permission to meet and collect data from their community. Verbal informed consent was obtained from participants before conducting the interview. The respondent's participation was voluntary where the respondents had the right to refuse the interview process.

Results

Table 1 shows the distribution of respondents accordingly to sociodemographic characteristics, the prevalence of knowledge and the use of modern contraceptive methods. Nearly half (46%) of the respondents were between 19-29 years, with an average age of 29 years. Over three-quarters of respondents (78.5%) had 0 to 2 living children, and almost half (48%) of respondents had primary level education. Approximately half (49.5%) of respondents were housewives, while 37% of respondent's husbands were involved in the agriculture sector. Over 6 in 10 respondents had >20000 NRs family monthly income. Almost two thirds (69.2%) of respondents had good

what is the mean of knowledge score?
what is the mean of age?

knowledge of modern contraceptive methods, and 47.3% of respondents used modern contraceptive methods. Injectable (43.4%) was the most used modern contraceptive, an implant (3.7%) was the least used contraceptive. Additionally, over 7 in 10 (71%) respondents ever visited a health facility for FP counselling (Table 1).

Table 1: Background characteristics and modern contraceptive methods knowledge and practices of Muslim women (N=400) in Nepal

**Variables**	**Category**	**Frequency**	**Percentage**
Age of women, Mean (SD) (29±8.74)	≤18 Years	34	8.5
	19-29 Years	184	46.0
	≥30 Years	182	45.5
Parity	0 to 2	314	78.5
	3 or above	86	21.5
Family types	Nuclear	145	36.3
	Joint	255	63.7
Women's education	Illiterate	108	27.0
	Primary	192	48.0
	Secondary & above	100	25.0
Women's occupation	Agriculture	158	39.5
	Daily wages worker	44	11.0
	Housewives	198	49.5
Husband's occupation	Agriculture	148	37.0
	Business and service	50	12.5
	Daily wages worker	82	20.5
	Foreign migrant worker	120	30.0
Family income (NRs)	≤20000	158	39.5
	>20000	242	60.5
Knowledge of modern contraceptive methods	Poor knowledge	123	30.8
	Good knowledge	277	69.2
Practices of modern contraceptive methods	Yes	189	47.3
	No	211	52.7
Contraceptive practices (n=189)	Condom	33	17.5
	Oral contraceptive	49	25.9
	Injectable	82	43.4
	Implant	7	3.7
	Intrauterine Contraceptive Device	10	5.3
	Female sterilisation	8	4.2
Ever visited HFs for FP	Yes	284	71.0
	No	116	29.0

Table 2 depicts the different sociodemographic variables and knowledge and use of modern contraceptive methods. Nearly three fourth (72.5%) of respondents belonging to a joint family had good knowledge of modern contraceptive methods. Over half (56.6%) of respondents belonging to the nuclear family practised modern contraceptive methods. Respondents were having secondary and above education have reported greater (56.0%) use of modern contraceptive methods. More than half (51.6%) of women who had good knowledge of modern contraceptive methods used modern contraceptives. Six in ten (60.3%) respondents who visited a health facility for FP counselling have used modern contraceptive methods (Table 2).

Table 2: Factors associated with knowledge and practices of modern contraceptive methods among Muslim women (N=400) in Nepal

Variables	Frequency (%)	Knowledge contraceptive methods		P value	Practice contraceptive methods		P value
		No (%)	Yes (%)		No (%)	Yes (%)	
**Women's age**							
≤18 years	34 (8.5)	12 (35.3)	22 (64.7)	0.805	15 (44.1)	19 (55.9)	0.580
19-29 years	184 (46.0)	57 (31.0)	127 (69.0)		99 (53.8)	85 (46.2)	
≥30 years	182 (45.5)	54 (29.7)	128 (70.3)		96 (52.7)	86 (47.3)	
**Family type**							
Joint	255 (63.8)	70 (27.5)	185 (72.5)	0.058	147(57.6)	108(42.4)	0.006
Nuclear	145 (36.3)	53 (36.6)	92 (63.4)		63(43.4)	82(56.6)	
**Parity**							
0-2	314 (78.5)	97 (30.9)	217 (69.1)	0.907	164(52.2)	150(47.8)	0.836
≥3	86 (21.5)	26 (30.2)	60 (69.8)		46(53.5)	40(46.5)	
**Women's education**							
Illiterate	108 (27.0)	35 (32.4)	73 (67.6)		68(63.0)	40(37.0)	
Primary	192 (48.0)	60 (31.3)	132 (68.8)		98(51.0)	94(49.0)	
Secondary and above	100 (25.0)	28 (28.0)	72 (72.0)	0.772	44(44.0)	56(56.0)	0.020
**Women's occupation**							
Housewives	198 (49.5)	48 (24.2)	150 (75.8)		103(52.0)	95(48.0)	
Agriculture	158 (39.5)	68 (43.0)	90 (57.0)		80(50.6)	78(49.4)	
Daily wages	44 (11.0)	7 (15.9)	37 (84.1)	<0.00	27(61.4)	17(38.6)	0.444
**Husband's occupation**							
Agriculture	148 (37.0)	53 (35.8)	95 (64.2)		79(53.4)	69(46.6)	

why the salary is cut off at 20000?
Why were the occupations divided as such?

Business and service	82 (20.5)	16 (19.5)	66 (80.5)		20(40.0)	30(60.0)	
Daily wages	50 (12.5)	15 (30.0)	35 (70.0)	0.078	40(48.8)	42(51.2)	0.123
Foreign	120 (30.0)	39 (32.5)	81 (67.5)		71(59.2)	49(40.8)	
**Income (monthly)**							
≤20000	158 (39.5)	53 (33.5)	105 (66.5)		78(49.4)	80(50.6)	0.311
>20000	242 (60.5)	70 (28.9)	172 (71.1)		132(54.5)	110(45.5)	
**Knowledge of modern contraceptive methods**							
Poor					76(61.8)	47(38.2)	0.013
Good					134(48.4)	143(51.6)	
**FP counselling at HF**							
No					85 (73.3)	31(26.7)	<0.001
Yes					125(39.7)	190(60.3)	

why remove FP counselling as IV to knowledge?

Table 3 illustrates the determinants of knowledge and the use of modern contraceptive methods. Women who belonged to the nuclear family (aOR=0.598; 95% CI: 0.38,0.95**)** had lower odds of knowing modern contraceptive methods than those living in the joint family. Women having an occupation in the agricultural sector (aOR=0.379; 95% CI: 0.22, 0.64) were less likely to be aware of modern contraceptive methods compared to housewives. Regarding the practice of modern contraceptive methods, women with primary (aOR=2.59; 95% CI: 1.43, 4.72), secondary and above education (aOR=4.41; 95% CI:2.02,9.63) had a significantly higher odds of practices of modern contraceptive methods compared to illiterate women. Women living in a nuclear family (aOR=2.24; 95% CI:1.40,3.59) had more than two-folds higher odds of modern contraceptive methods practices compared to their counterparts. Additionally, the practice of modern contraceptives was significantly higher among women having an occupation in the business and service sector (aOR=2.55; 95% CI: 1.17,5.56) compared to agriculture. Women having good knowledge of modern contraceptive methods (aOR=2.73; 95% CI: 1.66, 4.51) and women who ever visited a health facility for FP counselling (aOR=4.40; 95% CI: 2.58, 7.50) were more likely to practice modern contraceptive methods compared to those who had poor knowledge on modern contraceptives and those who have not visited HF for FP counselling respectively (Table 3).

Table 3: Determinants of knowledge and practices of modern contraceptive methods among Muslim women (N=400) in Nepal.

**Variables**	**Knowledge on modern contraceptive methods**				**Practice of modern contraceptive methods**			
	**COR 95% CI**	**p**	**AOR 95% CI**	**p**	**COR 95% CI**	**p**	**AOR 95% CI**	**p**
**Women's age**								
≥30 years	1.00		1.00		1.00		1.00	
≤18 years	0.77 (0.36,1.67)	0.514	0.61 (0.25,1.48)	0.276	1.41 (0.68, 2.95)	0.357	1.22 (0.50,3.01)	0.664
19-29 years	0.94 (0.60,1.47)	0.786	0.84 (0.49,1.44)	0.533	0.96 (0.64,1.45)	0.839	0.99 (0.58,1.69)	0.968
**Family type**								
Joint	1.00		1.00		1.00		1.00	
Nuclear	0.66 (0.42, 1.02)	0.059	**0.60 (0.38,0.95)**	**0.030**	1.77 (1.17, 2.67)	0.006	**2.24 (1.40,3.59)**	**0.001**
**Parity**								
0-2	1.00		1.00		1.00		1.00	
≥3	1.03 (0.61,1.73)	0.907	1.11(0.61,2.01)	0.728	0.95 (0.59,1.53)	0.836	1.22 (0.67,2.24)	0.511
**Women's education**								
Illiterate	1.00		1.00		1.00		1.00	
Primary	1.05 (0.64, 1.75)	0.836	0.86 (0.49,1.54)	0.620	1.63 (1.01, 2.64)	0.047	**2.59 (1.43, 4.72)**	**0.002**
Secondary and above	1.23 (0.68,2.23)	0.490	0.77 (0.36, 1.64)	0.495	2.16 (1.24, 3.77)	0.006	**4.41(2.02,9.63)**	**<0.001**
**Women's occupation**								
Housewives	1.00		1.00		1.00		1.00	
Agriculture	0.42 (0.27,0.67)	<0.001	**0.38 (0.22, 0.64)**	**<0.001**	1.06 (0.70,1.61)	0.795	1.46 (0.84,2.52)	0.179
Daily wages worker	1.69 (0.71,4.04)	0.237	1.61 (0.62,4.19)	0.327	0.68 (0.35,1.33)	0.263	0.57 (0.25,1.29)	0.175
**Husband's occupation**								
Agriculture	1.00		1.00		1.00		1.00	
Business and service	2.30 (1.21,4.37)	0.011	1.56 (0.68,3.59)	0.295	1.72 (0.90, 3.30)	0.104	**2.55 (1.17,5.56)**	**0.019**
Daily wages workers	1.30 (0.652,2.60)	0.455	0.91 (0.42,1.94)	0.802	1.20(0.70,2.06)	0.504	1.29 (0.58,2.85)	0.532
Foreign migrant	1.16(0.70,1.93)	0.570	0.81 (0.40,1.62)	0.554	0.79 (0.49,1.29)	0.343	1.00 (0.49,2.02)	0.989

**Income (monthly) in NRs**								
≤20000	1.00		1.00		1.00		1.00	
>20000	1.24 (0.81,1.91)	0.328	1.02 (0.56, 1.83)	0.958	0.81 (0.54,1.21)	0.311	0.67(0.36,1.21)	0.177
**Knowledge of modern contraceptive methods**								
Poor knowledge					1.00		1.00	
Good knowledge					1.73 (1.12, 2.66)	0.014	**2.73 (1.66, 4.51)**	**<0.001**
**Ever visited HF for FP counselling**								
No					1.00		1.00	
Yes					3.49(2.17,5.60)	<0.00	**4.40 (2.58,7.50)**	**<0.001**

Bold Significant at $p < 0{:}05$.

why doesnt the "ever visit to hospital" not included inside the regression.

The first sentence of the first paragraph is redundant with the first sentence on the second paragraph.

There is no discussion regarding religion issues and cultural issues that influence the uptake of Family planning. especially when there are acceptable level of knowledge among them.

Any potential influence from social circle? in law?

And how does the lack of accessibility influence them since many of themn are using the injectable form which needs HCP to insert.

Discussion

This study showed that two-thirds of Muslim women had knowledge, and two in five women practised modern contraceptive methods. Knowledge of modern contraceptive methods was low among the women working in agriculture and living in nuclear families. The practice of modern contraceptives was poor among women with no education, lived in a nuclear family, husbands working in the agriculture sector, women having poor knowledge on modern contraceptive methods, and who didn't receive FP counselling at health facility.

This study revealed that 69% of women had good knowledge of modern contraceptive methods. Past studies reported mixed results on knowledge of modern contraceptive methods in Nepal. For example, a previous study (2016) reported low (44%) knowledge on modern contraceptive methods among Muslim women in Nepal [31]. Another study showed relatively higher (94.5%) knowledge on modern contraceptive methods in Nepal [38]. About 87% of women knew contraceptive methods in India [39]. Exposure to FP information through mass media message dissemination, community HWs and Female Community Health Volunteers (FCHVs) in the study area might have helped acquire good knowledge on modern contraceptive methods.

Any different characteristics between ref 31, 34 , 40, 41, 42and this study?

Despite high proportion of good knowledge on modern contraceptive methods, Muslim women had low practices of modern contraceptive methods in Nepal. The religious beliefs, societal pressure and fear of going against religious values could be a potential driving force of lower practices of modern contraceptive methods [40]. Our study's finding is consistent with past studies conducted in Bangladesh [41], and India [42]. Injectable was the most practised modern contraceptive method, followed by oral contraceptive pills. Similar to our findings, previous research conducted in the eastern district of Nepal also reported injectables as the most used contraceptives (53.1%), followed by oral contraceptives (24%) [31]. Likewise, another study conducted in the Kapilbastu district in Nepal reported that injectable (51.3%) was the commonly used contraceptive method followed by oral contraceptive (25.6%) [19]. Injectables are the most preferred modern contraceptive methods among Muslim women in Nepal. Their popularity could be due to their simplicity, effectiveness for three months and accessibility even in private pharmacies at low cost [43].

The knowledge of modern contraceptive methods was influenced by several socioeconomic factors such as family type and occupation of women. The current study revealed that women

why is it that nuclear family has lower knowledge but higher practice than joint family? Does this infer cultural influence?

who lived in the nuclear family and were involved in the agricultural sector had poor knowledge of modern contraceptive methods. The women belonging to a nuclear family may have limited exposure to other family members, resulting in less opportunity to obtain information about contraceptive methods. The finding of this study is consistent with the study conducted in India [44]. However, previous studies in Nepal have reported no association between type of family and knowledge on modern contraceptive methods [31,38]. This could be because those women working in the agriculture sector might lack access to information on contraception

Several determinants such as education, good knowledge of contraceptive methods and access to counselling services were positively associated with the practices of modern contraceptive methods. Studies from Nepal [17,31] and other Asian countries [45] have reported increased practices of modern contraceptive methods with increased education [17,45]. The findings of the current study are consistent with the previous studies conducted in Nepal [31], Bangladesh [41], and India [42]. The illiterate women may have limited access to contraceptives, leading to a lack of awareness about the benefits of contraceptive use. Furthermore, those women may not openly discuss contraceptives with their spouse due to lower autonomy in marital relationships [21,46]. Previous evidence showed that illiterate Muslim women tended to become unaware of their reproductive rights and were very reluctant to visit health facilities for FP services [18].

Past evidence documented that having good knowledge of contraceptive methods may increase the practice of these contraceptives [21,47]. Our study also showed that women’s knowledge of modern contraceptive methods was related to their practice. Women who had good knowledge were more likely to practice modern contraceptive methods than those with poor knowledge. This might be because women with good knowledge may know better about the benefits of contraceptive use, and it would increase the women’s decision making power for the practice of contraceptives [48]. Moreover, access to FP counselling was another factor affecting contraceptive practices in our study; women who had ever visited a health facility for FP counselling were more likely to practice modern contraceptive methods than those who have not visited. The women who ever visited a health facility for FP counselling might be aware of the benefits of contraceptive use so, and they have favourable behaviour for the practices of contraceptive methods. A similar study conducted from abroad reported consistent findings [49].

Programmatic implications

what do you mean by gaps in knowledge?clarify

This study has highlighted some implications for policy and programs. First, the current study revealed gaps in good knowledge in modern contraceptive methods but poor practices of modern contraceptive methods. These women groups require accessible quality of contraceptive choices. The concept of roving midwives service providers can be adopted and implemented to offer counselling and FP services at doorsteps. Second, some targeted Social Behaviour Change Communication (SBCC) can improve the awareness of contraceptives, and practices include mass media mobilisation programs coherent with their religious values and promote them for FP services. Third, contraceptive practices can be improved through several demand and supply-side strategies. Supply-side approaches could be the recruitment of local HWs and FCHVs from the Muslim community. The local health workforce of the Muslim community can encourage them to practice contraceptives. In addition, there is a need for male engagement to inform Muslim men about the practice of contraceptives and change attitudes towards women's practice of different contraceptive options. Fourth, demand-side approaches could raise awareness and develop education materials in Urdu with Muslim women and work to extend support for smaller family norms, providing counselling and advice about contraceptive practice from the community and religious leaders. Other approaches such as mobile camps, satellite camps, and home visits could be essential to promote the contraceptive practice. Fourth, this study suggests that the existing blanket approach program may not address the Muslim community's issues. The Ministry of Health and Population (MOHP) should design program strategies based on a deeper understanding of needs, including religious and cultural recognition.

Strength and limitation of the study

dont see this being discussed. Seems like even primary health care is offering a few options of contraception. so why are Muslim women not forthcoming for FP? Any restriction by the healthcare system or political provision?

This study has some strengths. We have used pretested and well-designed questions and trained interviewers from the local community. The study has explored the factors influencing the practice of modern contraceptive methods among most unreached groups. However, this study has some limitations: First, it was a survey design that does not provide us with inferences regarding causality. Second, some of the important covariate such as distance to a health facility where FP service is available and cost that previous studies found important predictor of contraceptive practices were not included in this study [50,51]. Third, this study cannot be generalised to all populations as this study was conducted among Muslim women. Though this

study provided a cross-sectional analysis of knowledge and practices, a qualitative study can explore the underlying drivers of gaps in high knowledge and low practices of modern contraceptive methods among Muslim communities in Nepal.

Conclusions

The practice of modern contraceptive methods is relatively low despite having satisfactory knowledge among Muslim women. The poor knowledge and practice of modern contraceptive methods are seen especially among socially disadvantaged groups. To improve FP practices among Muslims in Nepal need integrated and focused health interventions. Such program interventions include culturally sensitive health education and information dissemination, SBCC interventions, mobilisation and home visits using local midwives, advice and supplies, and male mobilisers to reach out to Muslim men. The provision of FP services in mobile and satellite health camps, could improve the practices of FP services among Muslims in Nepal.

Abbreviation

FP-Family Planning

FPAN-Nepal Family Planning Association of Nepal

NFCC-Nepal Fertility Care Center

IUCD-Intrauterine Contraceptive Device

SBCC-Social Behavior Change Communication

HW-Health Workers

FCHV-Female Community Health Volunteers

NHSS-Nepal Health Sector Strategy

MOHP-Ministry of Health and Population

PHCC-Primary Health Care Center

Declaration

Conflict of interest

The authors declare that the research was conducted without any commercial or financial relationships that could be construed as a potential conflict of interest.

Funding

No funding received for this project.

Availability of data

The data of this study can be obtained upon request to first author.

Acknowledgement

Not applicable

Author Contribution

Conceptualisation: DST, YKC, RBK. Analysis: DST, YKC, RBK. Methodology: DST, YKC, RK, RBK. Original draft: DST, YKC, RK, RBK. Interpretation of data and revision of manuscript: DST, RBK. Revised and approved final version: DST, YKC, RK, and RBK.

References

1. Ganatra B, Faundes A. Role of birth spacing, family planning services, safe abortion services and post-abortion care in reducing maternal mortality. Best Pract Res Clin Obstet Gynaecol. 2016;36: 145–155. doi:10.1016/j.bpobgyn.2016.07.008

2. Chola L, McGee S, Tugendhaft A, Buchmann E, Hofman K. Scaling up family planning to reduce maternal and child mortality: The potential costs and benefits of modern contraceptive use in South Africa. PLoS One. 2015;10: 1–16. doi:10.1371/journal.pone.0130077

3. Abdi B, Okal J, Serour G, Temmerman M. "children are a blessing from God"- A qualitative study exploring the socio-cultural factors influencing contraceptive use in two Muslim communities in Kenya. Reprod Health. 2020;17: 1–11. doi:10.1186/s12978-020-0898-z

4. Mustafa G, Azmat SK, Hameed W, Ali S, Ishaque M, Hussain W, et al. Family Planning Knowledge, Attitude and Practices Among Married Men and Women in Rural Areas of Pakistan. Internatoinal J Reprod Med. 2015;2015: 8. doi:10.1155/2015/190520

5. Sundaram A, Hussain R, Sathar Z, Hussain S, Pliskin E, Weissman E. Adding It Up: Costs and Benefits of Meeting the Contraceptive and Maternal And Newborn Health Needs of Women in Pakistan. Guttmacher Inst. 2019. Available: https://doi.org/10.1363/2019.30703

6. Adhikari R, Soonthorndhada K, Prasartkul P. Correlates of unintended pregnancy among currently pregnant married women in Nepal. BMC Int Health Hum Rights. 2009;9: 1–10. doi:10.1186/1472-698X-9-17

7. Bekele D, Surur F, Nigatu B, Teklu A, Getinet T, Kassa M, et al. Knowledge and attitude towards family planning among women of reproductive age in emerging regions of ethiopia. J Multidiscip Healthc. 2020;13: 1463–1474. doi:10.2147/JMDH.S277896

8. Ajit Pradhan, Bal Krishna Suvedi, Sarah Banett, Sharad Kumar Sharma, Mahesh Puri, Pradeep Paudel, Shovana Rai Chitrakar, Naresh Pratap K.C. LH 2010. Nepal Maternal Mortality and Morbidity Study 2008/2009, Family Health Division Department of Health Services Ministry of Health and Population Government of Nepal Kathmandu, Nepal. 2010. Available: http://nnfsp.gov.np/PublicationFiles/aaef7977-9196-44d5-b173-14bb1cce4683.pdf

9. Bhandari A, Gordon M, Shakya G. Reducing maternal mortality in Nepal. BJOG An Int J Obstet Gynaecol. 2011;118: 26–30. doi:10.1111/j.1471-0528.2011.03109.x

10. Governement of Nepal. Family Planning in Nepal: Saving Lives and Improving Health. Ministry of Health and Population, Kathmandu. 2015. Available: https://www.healthpolicyproject.com/pubs/826_FamilyPlanningFINAL.pdf

11. Khatri RB, Durham J, Assefa Y. Utilisation of quality antenatal, delivery and postnatal care services in Nepal: An analysis of Service Provision Assessment. Global Health. 2021;17: 1–17. doi:10.1186/s12992-021-00752-x

12. Ministry of Health and Population (MOHP) [Nepal] New ERA and ICF International Inc. Nepal Demographic and Health Survey 2016,Kathmandu Nepal:Ministry of Health and Population, New ERA, and ICF International ,Calverton, Maryland: 2017.

13. Mehata S, Paudel YR, Dhungel A, Paudel M, Thapa J, Karki DK. Spousal Separation and Use of and Unmet Need for Contraception in Nepal: Results Based on a 2016 Survey. Sci

World J. 2020;2020. doi:10.1155/2020/8978041

14. Mehata S, Paudel YR, Mehta R, Dariang M, Poudel P, Barnett S. Unmet need for family planning in nepal during the first two years postpartum. Biomed Res Int. 2014;2014. doi:10.1155/2014/649567

15. Jabeen S, Rathor A, Riaz M, Zakar R, Fischer F. Demand- and supply-side factors associated with the use of contraceptive methods in Pakistan: a comparative study of demographic and health surveys, 1990–2018. BMC Womens Health. 2020;20: 1–11. doi:10.1186/s12905-020-01112-4

16. Alomair N, Alageel S, Davies N, Bailey J V. Factors influencing sexual and reproductive health of Muslim women: A systematic review. Reprod Health. 2020;17: 1–15. doi:10.1186/s12978-020-0888-1

17. Mehata S, Paudel YR, Dotel BR, Singh DR, Poudel P, Barnett S. Inequalities in the use of family planning in rural Nepal. Biomed Res Int. 2014. doi:10.1155/2014/636439

18. Arjyal A, Joshi D, Maharjan U, Regmi S, Baral SC, Marston C, et al. Access to family planning services by Muslim communities in Nepal – barriers and evidence gaps. 2016. Available: https://www.herdint.com/publications/9

19. Sapkota D, Adhikari SR, Bajracharya T, Sapkota VP. Designing Evidence-Based Family Planning Programs for the Marginalized Community: An Example of Muslim Community in Nepal. Front Public Heal. 2016;4: 1–10. doi:10.3389/fpubh.2016.00122

20. Suvedi B, Krishna A, Pradhan S, Barnett M, Puri SR, Chitrakar P, et al. Nepal Maternal Mortality and Morbidity Study, 2008/09. 2009.

21. Semachew Kasa A, Tarekegn M, Embiale N. Knowledge, attitude and practice towards family planning among reproductive age women in a resource limited settings of Northwest Ethiopia. BMC Res Notes. 2018. doi:10.1186/s13104-018-3689-7

22. Governement of Nepal. Nepal Health Sector Strategy.Ministry of Health and Population, Kathmandu. 2015. Available: https://nepal.unfpa.org/en/publications/nepal-health-sector-strategy-2015-2020

23. Ministry of Health and Population. Population Perspective Plan 2010-2031. 2010. Available: https://nepalindata.com/resource/population-perspective-plan-2010-2031-/

24. Shrestha DR, Shrestha A, Ghimire J. Emerging challenges in family planning programme in Nepal. J Nepal Health Res Counc. 2012;10: 108–112.

25. Governement of Nepal, Ministry of Helath and Population, Department of Health Services FHD. National Family Planning Costed Implementation Plan 2012-2015. 2015. Available: https://nepal.unfpa.org/en/publications/national-family-planning-costed-implementation-plan-2015-2020

26. Nepal G of. Annual Report: Department of Health Services 2075/76 (2019/19), Departement of Health Services, Ministry of Health and Population. 2019. Available: https://dohs.gov.np/

27. CBS. National Population and Housing Census 2011; National Planning Commission Secretariates, Central Bureau of Statistics. 2012. Available: http://cbs.gov.np/?p=2017

28. Bhusal CK, Bhattarai S. Factors Affecting Unmet Need of Family Planning Among Married Tharu Women of Dang District, Nepal. Int J Reprod Med. 2018;2018: 1–9.

doi:10.1155/2018/9312687

29. Dhungel S. Provincial Comparison of Development Status in Nepal: An Analysis of Human Development Trend for 1996 to 2026. J Manag Dev Stud. 2018;28: 53–68. doi:10.3126/jmds.v28i0.24958

30. Governement of Nepal. Health Management Information System (HMIS), Ministry of Health and Population. 2020. Available: http://hmis.gov.np/hmis/dhis-web-reporting/displayViewDocumentForm.action

31. Sigdel A, Alam M, Bista A, Sapkota H, Ghimire BS. Knowledge and utilization of family planning measures in a Muslim community of Sunsari district , Nepal. Imp J Interdiscip Res. 2016;2: 359–363.

32. Gizaw A, Regassa N. Family planning service utilization in Mojo town, Ethiopia: A population based study. J Geogr Reg Plan. 2011;4: 355–363. Available: http://www.academicjournals.org/JGRP

33. Bhatta DN, Haque A. Health problems, complex life, and consanguinity among ethnic minority Muslim women in Nepal. Ethn Heal. 2015;20: 633–649. doi:10.1080/13557858.2014.980779

34. Sharma D, Pokharel HP, Budhathoki SS, Yadav BK, Pokharel RK. Antenatal Health Care Service Utilization in Slum Areas of Pokhara Sub-Metropolitan City, Nepal. J Nepal Health Res Counc. 2016;14: 39–46.

35. Madjdian DS, Bras HAJ. Family, Gender, and Women’s Nutritional Status: A Comparison Between Two Himalayan Communities in Nepal. Econ Hist Dev Reg.

2016;31: 198–223. doi:10.1080/20780389.2015.1114416

36. Kassahun CW, Mekonen AG. Knowledge, attitude, practices and their associated factors towards diabetes mellitus among non diabetes community members of Bale Zone administrative towns, South East Ethiopia. A cross-sectional study. PLoS One. 2017;12: 1–18. doi:10.1371/journal.pone.0170040

37. Lincoln J, Mohammadnezhad M, Khan S. Knowledge, Attitudes, and Practices of Family Planning Among Women of Reproductive Age in Suva, Fiji in 2017. J Women's Heal Care. 2018;07: 3–8. doi:10.4172/2167-0420.1000431

38. Dhakal U, Shrestha RB, Bohara SK, Neupane S. Knowledge, Attitude and Practice on Family Planning among Married Muslim Women of Reproductive Age. J Nepal Health Res Counc. 2020;18: 238–242. doi:10.33314/jnhrc.v18i2.2244

39. Shumayla S, Kapoor S. Knowledge, attitude, and practice of family planning among Muslim women of North India. Int J Med Sci Public Heal. 2017;6: 1. doi:10.5455/ijmsph.2017.1266308122016

40. Boonstra H. Islam, women and family planning: A primer. Guttmacher Rep. 2001;December: 4–7.

41. Kamal SMM. Socioeconomic factors associated with contraceptive use and method choice in urban slums of Bangladesh. Asia-Pacific J Public Heal. 2015;27: NP2661–NP2676. doi:10.1177/1010539511421194

42. Suresh Sharma. Are Muslim women behind in their knowledge and use of contraception in India? J Public Heal Epidemiol. 2011;3: 632–641. doi:10.5897/jphe11.153

43. Karkee R, Adhikary S. A Cross-Sectional Survey of Contraceptive Use and Birth Spacing Among Multiparous Women in Eastern. Asia-Pacific J Public Heal. 2020. doi:10.1177/1010539520912117

44. Sindhu BM, Angadi MM. Knowledge, Attitude, and Practice about Family Planning Methods among Reproductive Age Group Women in a Tertiary Care Institute. Int J Sci Study. 2016;4: 133–136. doi:10.17354/ijss/2016/269

45. Najafi-Sharjabad F, Zainiyah Syed Yahya S, Abdul Rahman H, Hanafiah Juni M, Abdul Manaf R. Barriers of modern contraceptive practices among Asian women: a mini literature review. Glob J Health Sci. 2013;5: 181–192. doi:10.5539/gjhs.v5n5p181

46. Senarath U, Nalika Sepali Gunawardena. Women's autonomy in decision making for health care in South Asia. Asia-Pacific J Public Heal. 2009;21: 137–143. doi:10.1177/1010539509331590

47. Rodríguez E, Arqués JL, Rodríguez R, Nuñez M, Medina M, Talarico TL, et al. We are IntechOpen , the world ' s leading publisher of Open Access books Built by scientists , for scientists TOP 1 %. Intech. 1989;32: 137–144.

48. Dadi D, Bogale D, Minda Z, Megersa S. <p>Decision-Making Power of Married Women on Family Planning Use and Associated Factors in Dinsho Woreda, South East Ethiopia</p>. Open Access J Contracept. 2020;Volume 11: 15–23. doi:10.2147/oajc.s225331

49. Korra A 2002. Planning and Reasons for Nonuse among Women with Unmet Need for Family Planning in Ethiopia. Calverton, Maryl USA ORC Macro.

50. Gonie A, Wudneh A, Nigatu D, Dendir Z. Determinants of family planning use among married women in bale eco-region, Southeast Ethiopia: A community based study. BMC Womens Health. 2018. doi:10.1186/s12905-018-0539-7

51. Eliason S, Awoonor-Williams JK, Eliason C, Novignon J, Nonvignon J, Aikins M. Determinants of modern family planning use among women of reproductive age in the Nkwanta district of Ghana: A case-control study. Reprod Health. 2014;11: 1–10. doi:10.1186/1742-4755-11-65

Figure Click here to access/download;Figure;Figures.docx

Figure. 1

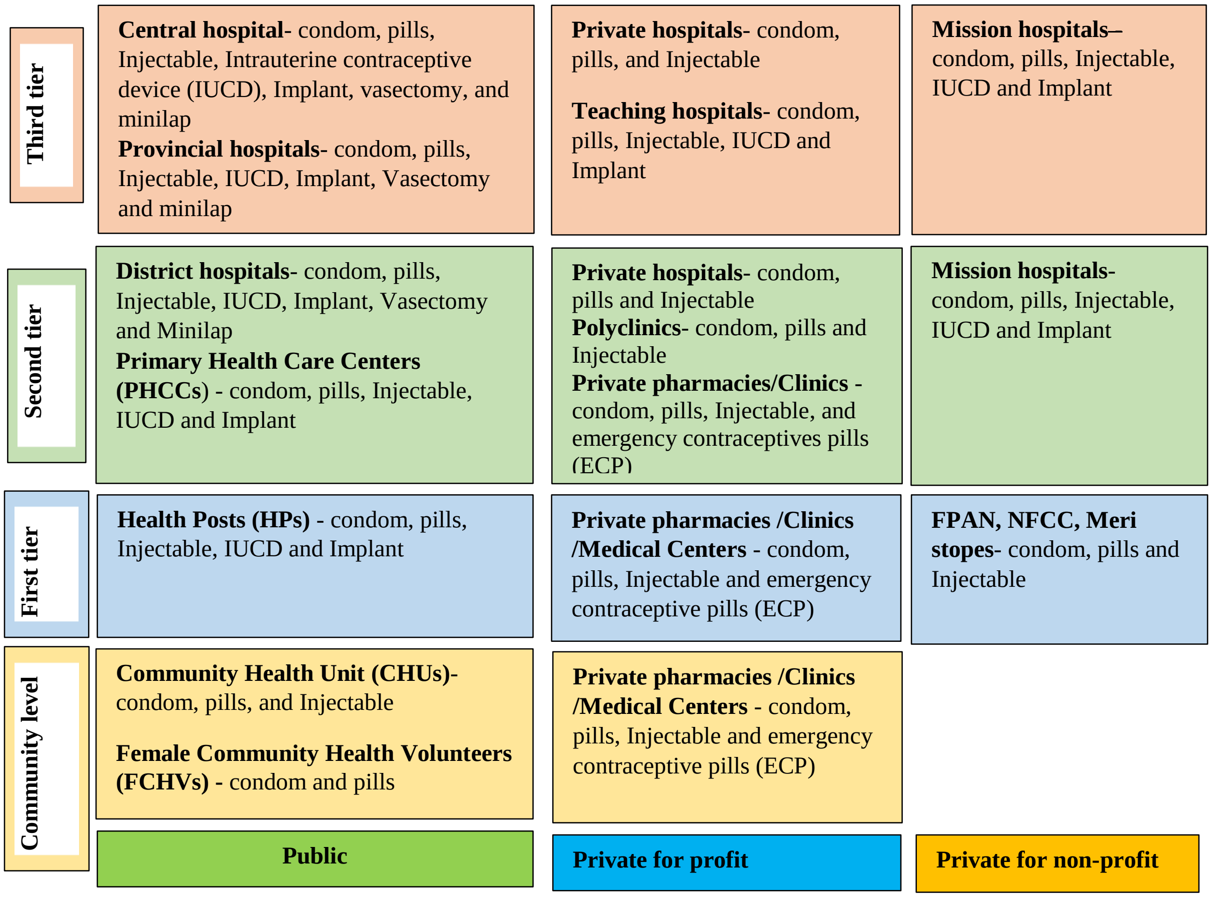

Figure. 2

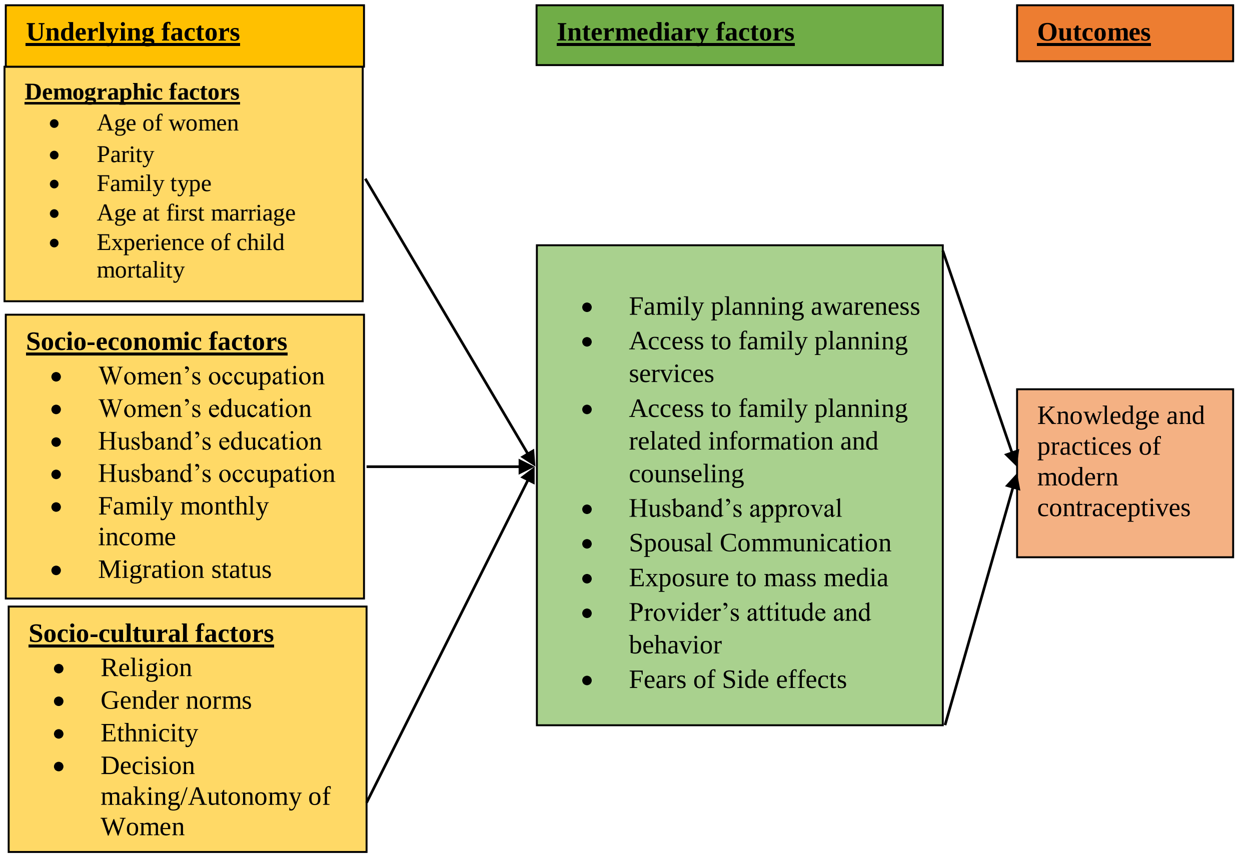
Underlying factors
Demographic factors
Age of women
Parity
Family type
Age at first marriage
Experience of child mortality
Socio-economic factors
Women's occupation
Women's education
Husband's education
Husband's occupation
Family monthly income
Migration status
Socio-cultural factors
Religion
Gender norms
Ethnicity
Decision making/Autonomy of Women
Intermediary factors
Family planning awareness
Access to family planning services
Access to family planning related information and counseling
Husband's approval
Spousal Communication
Exposure to mass media
Provider's attitude and behavior
Fears of Side effects
Outcomes
Knowledge and practices of modern contraceptives
