# Supplementary material for: Knowledge and practices of modern contraceptives among religious minority (Muslim) women: A cross-sectional study from Southern Nepal
Source: PLoS One. 2022 Dec 12;17(12):e0278899. doi: 10.1371/journal.pone.0278899 (PMC9744303; doi:10.1371/journal.pone.0278899)
Supplement: Attachment — Submitted filename: Response to Reviewers.doc [file pone.0278899.s003.doc]

June 5, 2022

Dear Editors-in-Chief

PLOS ONE

Thank you very much for your email with the decision dated April 26, 2022. We found that reviewers' and editor feedbacks were insightful. We have addressed both reviewers’ and editors' comments point by point. We have uploaded the revised manuscript showing track changes so that you can see all revisions and modifications we have made. We believe that our revisions will satisfy you and both reviewers. We have included a clean copy and track change copy of the revised manuscript.

Thank you for considering this manuscript for publication.

Sincerely,

Dipendra Singh Thakuri

On behalf of Yamuna KC Singh, Rajendra Karkee, Resham Bahadur Khatri

Editor

1. Was the knowledge score normally distributed? What was the cut off score for the good and poor knowledge? Please provide the results of knowledge score in mean, median and the minimum and maximum score.

***Authors response:*** *Thank you so much for your insightful comments. The knowledge score was not normally distributed; therefore, we employed to run logistic regression to identify the factors associated with the outcome variables of this study. We dichotomised the knowledge and practices scores based on previous studies using mean cut-off point. Below the mean score, we consider as poor knowledge and mean and above as good knowledge. Additionally, we have added results of knowledge scores in mean, median and the maximum and minimum scores in the page no. 9 of revised manuscript.*

2. Table 2 Kindly change the No and yes for knowledge contraceptive methods to poor (score <xx) and good (score >= xxx).

***Authors response****: Thank you so much for your comments. We have changed it in the revised manuscript as suggested.*

3. Table 3 Title: kindly specify determinants of good or poor knowledge

***Authors response****: Thank you so much for your comments. We have revised it as suggested in the revised manuscript. “The good and poor knowledge was determined by using mean cut-off point. We considered below the mean score as poor knowledge and mean and above as good knowledge.”*

4. The suggestion in implication need to base on the results. This study did not show any results of male engagement, thus might not appropriate to introduce this in the pragmatic implications and conclusion.

***Authors response****: Thank you so much for your important feedback. We agree with you, and we have removed it?*

Reviewer #1

1. Abstract: Data were collected using face-to-face semi-structured interviews. Semi-structured is for qualitative. Remove semi.

***Authors response****: Thank you for your suggestion. It was a typo error. We have corrected it.*

2. In Nepal, modern contraceptive services are provided from different outlets ranging from community to tertiary level (Figure 1). If this is, what is the real gap in your study?

***Authors response****: Thank you so much for your comment. Despite the availability of FP services, our study population does not go or is very reluctant to take services from local health posts and health centres.*

3. Your method part is shallow; try to address the study period, data analysis...

***Authors response****: Thank you so much for your comments. Our study period was between June and September 2019, described under the study design and setting section on page no. 6. Similarly, we have mentioned details about data analysis under the separate data analysis section on page no.8.*

4. Page- 5: Method part, 1st paragraph “Muslims in Nepal”. This is unclear. What is its importance? This sensitive issue (religious-based). Better if it’s addressed in the statement of the problem as a gap by linking with your objectives.

***Authors response:*** *Thank you so much for your important feedback. We have reviewed and moved the information to the Introduction part.*

5. Page 5: Your sample size is 417, i.e., p-0.44 + 10% non-response rate= 417. How have you said 400?

***Authors response****: Thank you so much for your important comment. Non-response rate has been reported to be very low by various studies in Nepal, so we take a 5% non-response rate. Considering a 5% non-response rate, we calculated a sample size of 398, So we collected 400 as our final sample size. We have taken the reference of past studies to consider the non-response rate of 5%.*

<https://www.jnhrc.com.np/index.php/jnhrc/article/view/2244/939>

6. Page 6: Conceptual framework of the study. What is the importance? i.e. its repetition you have already in the discussion part. If it has justifiable importance, take it at the end of the introduction rather than in the method part.

***Authors response****: Thank you so much for your valuable comment. We have removed it as you suggested.*

7. Page 6: “Study variables”. Minimize it- put the main list of the variables.

***Authors response****: Thank you so much for your suggestions. We have revised it as suggested.*

8. Page 6: Outcome variables. Similarly, minimize it- as good/poor knowledge of modern contraceptive methods is assessed from 10 items and then we considered a score of mean and above ‘Good Knowledge’ and a score of below mean ‘poor knowledge [7, 36]. Do not list everything.

***Authors response****: Thank you so much for your important feedback. We have revised it as suggested. Please see it in the page no. 7 of revised manuscript.*

9. Adopted–adaptation
10. Result part: add the descriptive finding of 10 sets of questions related to knowledge about modern contraceptives.

***Authors response****: Thank you so much for your important comments. We have added descriptive findings of 10 sets of questions in the revised manuscript*, see table 2 of the revised manuscript*.*

11. It needs an English edition

***Authors response****: Thank you so much for your comment. We have reviewed and edited the English language in the revised manuscript.*

Reviewer #2:

This article has potential for highlighting social, religion and cultural issue in family planning among the minority ethnic group of women.
Introduction: The objectives are not clear. Investigating the status of knowledge and practice need clarification. The justification and problem gaps were not clearly laid down. Why still study the practice status when there have been studies that demonstrated the low CPR among Nepal minority group?

***Authors response****: Thank you so much for your comment. We have revised objectives and added information about problem gaps and justification in the introduction section*.

Methods:
Information regarding the venue not clear- work office? Was it done from the hospital base or the central office of MWRA community?

***Authors response****: Thank you so much for your comment. It was a community based study, and data were collected at a community level. A face to face interview was carried out in the respondent’s household. We have described it in the methodology part on page no.8 of the revised manuscript.*

Sample size calculation needs to demonstrate a recalculation that is based on the two dependent variables (not only practice base). I would also advise to use the two proportion sample size formula.

***Authors response****: Thank you so much for your insightful comments /suggestions. We appreciate your important suggestion. We considered practice as our main outcome variable, so we only considered sample size calculation using practice prevalence. We have taken the reference of past studies where a sample was calculated based on the one dependent variable (Practice).* <https://bmchealthservres.biomedcentral.com/articles/10.1186/s12913-018-3643-3>

<http://pubs.sciepub.com/ajphr/5/1/1/>

Why is the salary cut off at 20000? Why were the occupations divided as such?

***Authors response:*** *Thank you so much for your comment. We have taken reference of previously published literature for salary cut off and dividing occupations*.

<https://www.ncbi.nlm.nih.gov/pmc/articles/PMC6510098/>

<http://www.aimspress.com/article/10.3934/publichealth.2019.3.291>

Why doesn’t the "ever visit to hospital" not included inside the regression?

***Authors response****: Thank you so much for your comment. We have one variable regarding “ever visited a health facility for FP counselling” that we included inside the regression. Besides that, we didn’t have variables like ever visited hospital in this study, so we couldn’t include it.*

Discussion/ conclusion: Since this study was done among a minority Muslim woman; unfortunately, there is very little said regarding the role of religion and culture in the family planning practice.

***Authors response****: Thank you so much for your comments. We have added details regarding the role of religion and culture in the revised manuscript (see the introduction, pages 3 and 4).*

Why would there be a low practice despite an acceptable percentage of women with good knowledge. The author earlier mentioned about the inaccessible family planning services and also being the minority ethnic group how does this influence the status of family planning knowledge and practice, but nothing was discussed on thesis.

***Authors response:*** *Thank you so much for your comment. This mismatch between knowledge and practice shows knowledge always does not translate to practice because of other barriers, for example, cultural and economic reasons. We have discussed it in “Discussion”.*

Any different characteristics between ref 31, 40, 41, 42 and this study?

***Authors response***: *Thank you. These are studies from different settings, i.e., Nepal, India, Bangladesh, and other countries. Ref. 31 is a study conducted among Muslim women from eastern Nepal. Furthermore, Ref. 40-42, similar studies from abroad (India, Bangladesh, and other countries), so the context of these studies differs from this study.*

This article still needs English editing

***Authors response****: thank you so much for your suggestion. We have reviewed and edited English.*

We would like to thank editor and both the reviewers for their insightful comments and feedback. Thank you so much for inviting us to revision of this manuscript.

Dipendra Singh Thakuri, on behalf of all co-authors
