# Supplementary material for: Knowledge and practices of modern contraceptives among religious minority (Muslim) women: A cross-sectional study from Southern Nepal
Source: PLoS One. 2022 Dec 12;17(12):e0278899. doi: 10.1371/journal.pone.0278899 (PMC9744303; doi:10.1371/journal.pone.0278899)
Supplement: Attachment — Submitted filename: Response to reviewers.docx [file pone.0278899.s004.docx]

October 14, 2022

Dear Editors-in-Chief

PLOS ONE

Thank you very much for your email with the decision dated Aug 12, 2022. We found the reviewers' and editor's feedback and comments insightful. We have carefully addressed both reviewers’ and editor’s comments point by point. To facilitate your review, we have uploaded the revised manuscript in track change and clean versions so that you can see all revisions and modifications we have made. We believe that our revisions will satisfy you and both reviewers.

Thank you in advance for considering this manuscript for publication.

Sincerely,

Dipendra Singh Thakuri

On behalf of all co-authors

**Additional Editor Comments:**

Abstract
Conclusion
Suggest remove ‘Such interventions include mobilisation of health workers (HWs) from their community, and awareness of contraceptive methods embedding with values and culture of the Muslim religion.’ This would be more appropriate in discussion section.

***Authors' response****: Thank you so much for your insightful suggestions. We've moved it from the conclusion section to the discussion section. (Page # 19)*

Methodology
You have mentioned that the knowledge score is not normally distributed, if it is so, it might be more appropriate to use the median as the cut-off point. I can see that the mean and median are close i.e 6.9 vs 7, it is likely to be normally distributed, could you please check.

***Authors response***: Thank you so much for your important suggestion. We have used median as the cut-off point as advised.

Table 3:

Title: From your explanation and interpretation of the results, it looks like your interest group for your knowledge outcome is ‘good knowledge’. If it so, the title should be determinants of good knowledge…., so the aOR less than 1 is considered as having poorer knowledge. Please check your interpretation and your analysis.
For value that <0.00, please change to <0.001. Kindly check through all values

***Authors response:*** *Thank you so much for your great comments. We have reviewed and revised it as advised. For a value that <0.00, we have changed it in the revised manuscript.*

Discussion
First paragraph: Please check the interpretation and the facts for those lived in nuclear family in terms of their knowledge and practice.
It is worth to add in a paragraph to discuss about those who lived in nuclear family regarding their poor knowledge but higher practice of FP.

***Authors response:*** *Many thanks for your suggestions. We have addressed this in the discussion part of the revised manuscript. (Page # 18-19)*

Pragmatic implications
Could you please focus your intervention based on your results i.e. targeted the factors that could improve your practice based on your results (which is how to increase the knowledge in Muslim women, and intervention that could address this factor ‘ever visited a HF for HP counselling’).
The following facts need to be reorganised and relate it to the factors that you have found to influence your practice.
“These women groups require accessible quality of contraceptive choices. The concept of roving midwives service providers can be adopted and implemented to offer counselling and FP services at doorsteps. Second, some targeted Social Behaviour Change Communication (SBCC) can improve the
awareness of contraceptives, and practices include mass media mobilisation programs coherent with their religious values and promote them for FP services. Third, contraceptive practices can be improved through several demand and supply-side strategies. Supply-side approaches could be the recruitment of local HWs and FCHVs from the Muslim community. The local health workforce of the Muslim community can encourage them to practice contraceptives. Fourth, demand-side approaches could raise awareness and develop education materials in Urdu for Muslim women and work to extend support for smaller family norms, providing counselling and advice about the contraceptive practice from the community and religious leaders. Other approaches such as mobile camps, satellite camps, and home visits could be essential to promote the contraceptive practice. Lastly, this study suggests that the Ministry of Health and Population (MOHP) should design targeted program strategies for Muslim women based on a deeper understanding of needs, including religious and cultural recognition”

***Authors' response:*** *Thank you so much for your valuable comments. We have reviewed and revised this section based on our study findings.* (Page# 19)

Conclusion
The following would need to reorganise or rephrase after you have tidied up the discussion on the section of pragmatic implications: ‘To improve FP practices among Muslims in Nepal needs integrated and focused health interventions. Such program interventions include health education and information dissemination, SBCC interventions, mobilisation and home visits using local midwives, advice and supplies, and male mobilisers to reach out to Muslim men. In addition, the provision of FP services in mobile and satellite health camps could improve the practices of FP services among Muslims in Nepal.’

***Authors response***: *Thank you for your comment. We have revised it based on the revision made in the* discussion on the section on pragmatic implications*. (Page# 20)*

**Reviewer #1:**

Thank you, the author, for your effort. The previously raised comments are addressed well. On the methods and all via out the manuscript.

***Authors response:*** We thank the reviewer for their great comments.

**Reviewer #2:**

Hi;
The Manuscript is much improved. However, there are still a few sentences that have typo error and grammatically not correct. Some of the sentences are hanging. SO still need some work on English editing

**Authors response**: *Thank you so much for your comments. We have checked the typo error and grammar throughout the manuscript and edited English.*

2. There are 2 tables 3; need to redefine
3. Table 3 is cute crowded; suggest separating the factors associated with knowledge and factors associated with practice. The narrative part can be improved by this separation.

***Authors response****: Thank you so much for pointing this out. We have addressed this in the revised manuscript. Also, we have separated the knowledge and practice table as suggested, and the narrative part was revised accordingly (Page# 14-16)*

4. The interpretation of table 4 regarding nuclear family predicting contraceptive use is inaccurate.

***Authors response****: Thank you for your comment. We have rechecked the interpretation of table 4 regarding nuclear family and contraceptive use, and it seems fine.*

The discussion; sentence 4:
The practice of modern contraception is poor among women who lived in a nuclear family. But the OR for nuclear family in practising FP is 4.4

***Authors response:*** *Thank you so much for your important comment. It was a typo error. The women who lived in a nuclear family had good FP practice. We have corrected it in the revised manuscript. (Page #17)*

5.I feel that there is a lot to look at on how we can target women coming from nuclear family that can be elaborated.

***Authors' response****: We have added some recommendations regarding this in the programmatic implication section of the revised manuscript.*

6. Conclusion: some of the conclusions do not come from the data. such as the male mobiliser or cultural factors.

***Authors response:*** *Thank you so much for your comments. We have revised the conclusion as advised. Page #20)*

We want to thank the editor and both reviewers for their insightful comments and feedback. Thank you so much for inviting us to revise this manuscript.

Dipendra Singh Thakuri, on behalf of all co-authors
